# Associations of Biomarkers of Kidney Tubule Health, Injury, and Inflammation with Left Ventricular Hypertrophy in Children with CKD

**DOI:** 10.34067/KID.0000000000000183

**Published:** 2023-06-12

**Authors:** Kuan Jiang, Jason H. Greenberg, Alison Abraham, Yunwen Xu, Jeffrey R. Schelling, Harold I. Feldman, Sarah J. Schrauben, Sushrut S. Waikar, Michael G. Shlipak, Nicholas Wettersten, Steven G. Coca, Ramachandran S. Vasan, Orlando M. Gutierrez, Joachim H. Ix, Bradley A. Warady, Paul L. Kimmel, Joseph V. Bonventre, Chirag R. Parikh, Mark M. Mitsnefes, Michelle R. Denburg, Susan Furth

**Affiliations:** 1 Yale School of Medicine, New Haven, Connecticut; 2 University of Colorado, Anschutz Medical Campus, Denver, Colorado; 3 Johns Hopkins University, Baltimore, Maryland; 4 Case Western Reserve University, Cleveland, Ohio; 5 Perelman School of Medicine at the University of Pennsylvania, Philadelphia, Pennsylvania; 6 Boston University School of Medicine, Boston, Massachusetts; 7 University of California San Francisco, San Francisco, California; 8 University of California San Diego, San Diego, California; 9 Icahn School of Medicine at Mount Sinai, New York, New York; 10 University of Alabama at Birmingham, Birmingham, Alabama; 11 Children's Mercy Kansas City, Kansas City, Missouri; 12 NIDDK, Bethesda, Maryland; 13 Brigham and Women's Hospital, Boston, Massachusetts; 14 Cincinnati Children's Hospital Medical Center, Cincinnati, Ohio; 15 Children's Hospital of Philadelphia, Philadelphia, Pennsylvania

**Keywords:** CKD, children, chronic inflammation, echocardiography, glomerular disease, kidney tubule, left ventricular hypertrophy, pediatric nephrology, pediatrics, renal injury

## Abstract

**Key Points:**

- Higher plasma and urine kidney injury molecule-1, urine monocyte chemoattractant protein-1, and lower urine alpha-1-microglobulin were associated with left ventricular hypertrophy, even after adjustment for confounders.
- Biomarkers of tubular injury, dysfunction, and inflammation may indicate the severity of kidney pathology and are associated with left ventricular hypertrophy.

**Background:**

Left ventricular hypertrophy (LVH) is common in children with CKD and is associated with an increased risk of cardiovascular disease and mortality. We have shown that several plasma and urine biomarkers are associated with increased risk of CKD progression. As CKD is associated with LVH, we sought to investigate the association between the biomarkers and LVH.

**Methods:**

In the CKD in Children Cohort Study, children aged 6 months to 16 years with an eGFR of 30–90 ml/min per 1.73 m^2^ were enrolled at 54 centers in the United States and Canada. We measured plasma biomarkers kidney injury molecule-1 (KIM-1), tumor necrosis factor receptor-1, tumor necrosis factor receptor-2, soluble urokinase-type plasminogen activator receptor and urine KIM-1, monocyte chemoattractant protein-1 (MCP-1), YKL-40, alpha-1-microglobulin (alpha-1m), and epidermal growth factor in stored plasma and urine collected 5 months after enrollment. Echocardiograms were performed 1 year after enrollment. We assessed the cross-sectional association between the log_2_ biomarker levels and LVH (left ventricular mass index greater than or equal to the 95th percentile) using a Poisson regression model, adjusted for age, sex, race, body mass index, hypertension, glomerular diagnosis, urine protein-to-creatinine ratio, and eGFR at study entry.

**Results:**

Among the 504 children, LVH prevalence was 12% (*n*=59) 1 year after enrollment. In a multivariable-adjusted model, higher plasma and urine KIM-1 and urine MCP-1 concentrations were associated with a higher prevalence of LVH (plasma KIM-1 prevalence ratio [PR] per log_2_: 1.27, 95% confidence interval [CI], 1.02 to 1.58; urine KIM-1 PR: 1.21, 95% CI, 1.11 to 1.48; and urine MCP-1 PR: 1.18, 95% CI, 1.04 to 1.34). After multivariable adjustment for covariates, lower urine alpha-1m was also associated with a higher prevalence of LVH (PR: 0.90, 95% CI, 0.82 to 0.99).

**Conclusions:**

Higher plasma and urine KIM-1, urine MCP-1, and lower urine alpha-1m were each associated with LVH prevalence in children with CKD. These biomarkers may better inform risk and help elucidate the pathophysiology of LVH in pediatric CKD.

## Graphical abstract

**Figure:**
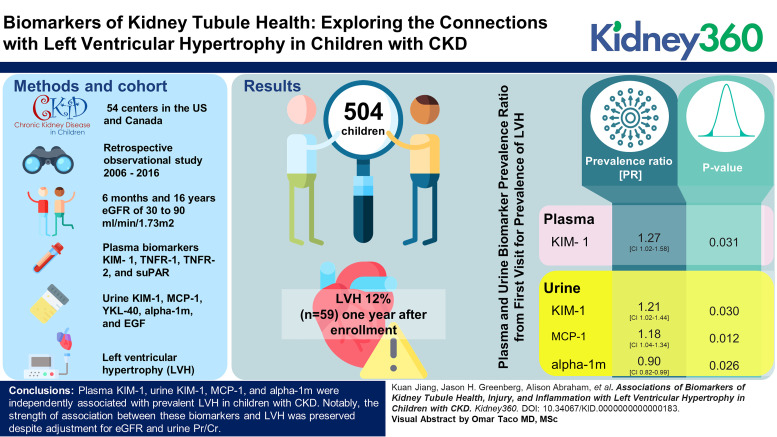

## Introduction

Cardiovascular disease (CVD) is highly prevalent in children with CKD. As CKD progresses and kidney function declines in children, the risk of CVD increases. Young adults with a history of childhood onset ESKD have as much as a 10–100-fold increased risk of coronary artery calcification, carotid arteriopathy, and left ventricular hypertrophy (LVH), compared with the general population.^1^ LVH, an increase in left ventricular mass (LVM), is an adaptive response to a chronically increased workload.^1^ LVH is considered an early or intermediate marker of CVD because it is strongly associated with an increased risk of future cardiovascular morbidity and mortality.^2^ As kidney function declines, the occurrence of LVH in children increases.^3^ In multiple studies, the prevalence of LVH in children with CKD was approximately 10%–35% of the overall study population.^2,4–7^

We have hypothesized that many of the biomarkers that are associated with CKD progression may also be associated with LVH development because CKD is strongly associated with LVH. Biomarkers of tubular injury, tubular dysfunction, and inflammation could potentially provide additional insight beyond traditional biomarkers, such as serum creatinine and proteinuria, as we strive to understand the pathophysiology of LVH in children with CKD. Kidney biomarkers may be used to characterize tubular health which plays a key role in the excretion of salt and water. Impaired salt and water excretion can lead to volume expansion, an increase in cardiac output, hypertension (HTN), and LVH.^8^ Furthermore, the reduced nephron mass and poor tubular health of CKD can activate the renin angiotensin system, adrenergic system, sympathetic nervous system, and cause endothelial dysfunction, leading to an increased risk of HTN and LVH.^9^ Currently, there is a paucity of research on biomarkers of LVH in children with CKD. In a study of children with CKD by Mitsnefes *et al.*, plasma fibroblast growth factor 23 (FGF23) was associated with a two-fold greater risk of LVH.^10^ Because CVD affects the long-term morbidity and mortality of children with CKD, a better understanding of LVH is critical to elucidate its pathophysiology and discover effective treatments.^3^

In prospective cohort studies of children with CKD, plasma biomarkers of tubular injury (kidney injury molecule-1 [KIM-1]), inflammation (tumor necrosis factor receptor [TNFR]-1, TNFR-2, soluble urokinase-type plasminogen activator receptor [suPAR]), and urine biomarkers of tubular injury (KIM-1), tubular dysfunction (alpha-1-microglobulin [alpha-1m]), tubular health (epidermal growth factor [EGF]), and inflammation (monocyte chemoattractant protein-1 [MCP-1], YKL-40) were found to be associated with CKD progression.^11,12^ In this present investigation, we analyzed these plasma and urine biomarkers in the CKD in children (CKiD) multicenter cohort study to determine their associations with LVH.

## Methods

### Study Participants

Participants were from the CKiD cohort, a study of children with CKD from 54 medical centers in the United States and Canada from 2006 through 2016.^13^ The children had a baseline eGFR of 30–90 ml/min per 1.73 m^2^ and were between the ages of 6 months and 16 years at study entry. Children who had a history of kidney, solid organ, or bone marrow transplantation; dialysis within 3 years of diagnosis; or a history of cancer were excluded from enrollment. Assent and written informed consent were provided by children and their parents or legal guardians, respectively. The CKiD study received approval by each participating organization's Institutional Review Board and has been registered with the identifier NCT00327860 on ClinicalTrials.gov. For our study, we included CKiD participants if they had sufficient plasma and urine volume stored and data on eGFR, urine protein-to-creatinine ratio (urine Pr/Cr), BP, glomerular diagnosis (GD), and body mass index (BMI) at study entry, as well as an echocardiographic assessment performed 1 year after study enrollment.^12^

### Plasma and Urine Biomarker Measurements

The exposures included plasma biomarker and urine biomarker levels indexed to urine creatinine. Plasma and urine samples were collected from participants a median of 5 months after CKiD study enrollment. All urine specimens used for biomarker measurements were from spot urine collections. Stored plasma and urine biospecimens were centrifuged and supernatents were aliquoted. The aliquots were labeled with a barcode and were stored at -80°C until the biomarker assays were measured in a central, clinical outcome-blind laboratory. All biomarkers were measured in a batch, and all measurements were performed in the Brigham and Women's Hospital Central Biomarker Consortium Laboratory.

Eight biomarkers were selected based on a literature review of CKD biomarkers and previous studies within the CKD Biomarker Consortium with consideration for mechanisms of CKD progression. A Meso Scale Discover assay was used to measure plasma KIM-1, TNFR-1, TNFR-2, and suPAR (Meso Scale Discovery, Gaithersburg, MD). A Luminex multiplex assay (Luminex Corporation, Austin, TX) was used to measure urine EGF, KIM-1, MCP-1, and YKL-40. All assays were conducted in duplicate, and the mean values for each biomarker were used in the analysis. A Siemen's nephelometer (Siemens, Munich, Germany) was used to measure urine alpha-1m. All intra-assay and inter-assay coefficients of variation were <10% (Supplemental Table 1).

Values below the limits of detection for urine biomarkers (KIM-1: 27.43 pg/ml, MCP-1: 5.49 pg/ml, YKL-40: 65.84 pg/ml, EGF: 6.86 pg/ml, alpha-1m: 5.64 mg/ml) and plasma biomarkers (KIM-1: 1.98 pg/ml, TNFR-1: 0.667 pg/ml, TNFR-2: 0.168 pg/ml, suPAR: 53 pg/ml) were imputed based on a lognormal distribution. This imputation method, truncated lognormal imputation, uses the distribution of observed data to recreate the lower tail of the distribution and then randomly draws a value to replace the missing information below the limit of detection.^12^ Nondetectable values were observed in 171, 156, 2, and 1 participant(s) for urine alpha-1m, urine YKL-40, urine MCP-1, and urine KIM-1, respectively. Plasma biomarkers and indexed urine biomarker values were considered continuous variables after transforming to log_2_ in analyses.

### Cardiovascular Outcomes from Echocardiograms

The outcome was LVH determined based on echocardiographic assessments conducted 1 year after enrollment in the CKiD cohort. M-mode and Doppler echocardiography were conducted at individual participating centers. Reading and analysis were performed by the Cardiovascular Core Imaging Research Laboratory at Cincinnati Children's Hospital Medical Center. LVH was defined as a LVM index (LVMI) ≥95th percentile for healthy children, and LVM was measured by two-dimensional directed M-mode echocardiography at rest according to the American Society of Echocardiography.^2,14,15^ LVM was indexed to height (mass [g]/height [m^2.7^]) to account for body size, but because LVMI does not adequately account for changes due to growth, we also expressed LVMI as Z-scores on the basis of age and sex.^14,16^ To have standardization and uniformity among the echocardiographic images across the centers, the qualifying recordings were sent to the Cardiovascular Core Imaging Research Laboratory.^2^

### Clinical and Laboratory Covariates

BMI was standardized for age and sex. HTN was defined as a systolic or diastolic BP ≥95th percentile for height, age, and sex. Glomerular and nonglomerular distinction was made for classification of kidney disease.^17^ We calculated eGFR on the basis of serum creatinine, cystatin C, and blood urea nitrogen concentrations from the modified Schwartz equation which is derived from the CKiD population.^18^ All serum creatinine measurements were performed in the CKiD central laboratory at the University of Rochester.

### Statistical Analysis

The primary analysis, a cross-sectional association of log_2_-transformed plasma and urine biomarker levels with prevalence of LVH, was estimated using a Poisson regression, with adjustment for demographics (age, sex, and race) and clinical variables (BMI, HTN, GD [yes/no], urine Pr/Cr, and eGFR at study entry). Two nested models were used to assess the influence of key clinical CKD factors on biomarker relationships: one partially adjusted model included age, sex, race, BMI, HTN, and GD and a second fully adjusted model that additionally included eGFR and urine Pr/Cr measured at study enrollment. In a secondary analysis, we assessed the cross-sectional association between log_2_-transformed plasma and urine biomarker levels and LVMI Z-score. Generalized estimating equations were used for a robust estimation of the error variance to account for potential overdispersion. Subgroup analysis by glomerular versus nonglomerular CKD status evaluated the potential heterogeneity of biomarker relationships by diagnosis. The presence of effect modification was tested by adding an interaction term of biomarker and diagnosis of glomerular CKD to models. Significant associations were recognized with *P* values <0.05. Analyses were performed using SAS 9.4 for Windows (SAS Institute Inc., NC) and R (R Core Team version 3.5.1).

## Results

### Study Participants

**Figure 1.**
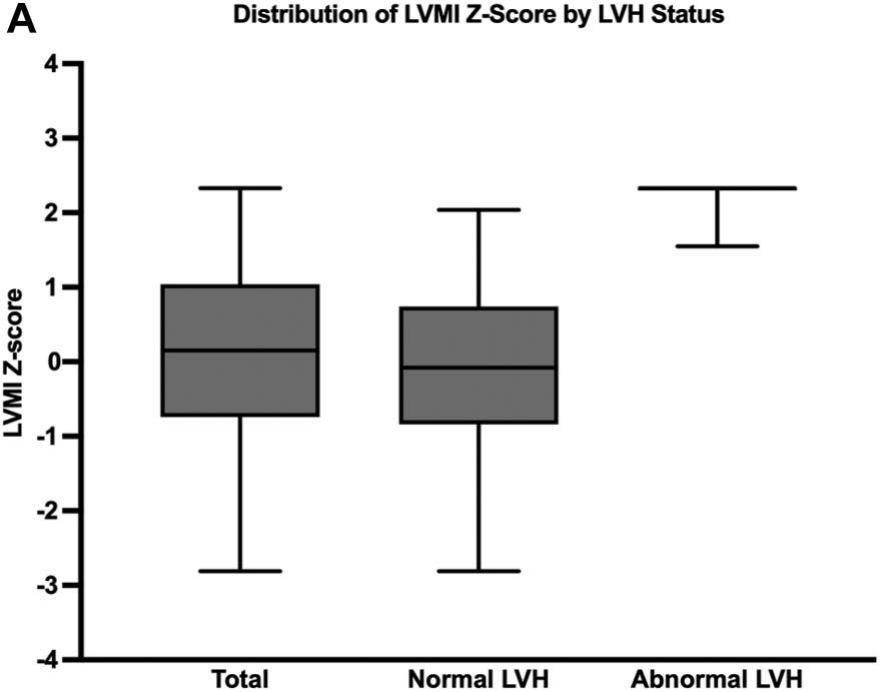
**Echocardiogram data were taken during the second visit, 6 months after their first visit.** Box plots show mean, upper and lower quartiles, and range of LVMI (A) for all enrolled participants, children without LVH, and children with LVH. LVH, left ventricular hypertrophy; LVMI, left ventricular mass index.

Of the 619 children with available biomarker measurements, 115 were excluded due to missing echocardiographic assessments, leaving 504 in the final analyses (Figure 1). The median age was 12 years (interquartile range [IQR], 8–15), 311 (62%) were male, 94 (19%) were Black, 67 (13%) were Hispanic, 156 (31%) had a glomerular cause of CKD, 85 (17%) had HTN, median baseline eGFR was 54 (IQR, 41–68) ml/min per 1.73 m^2^, and median urine Pr/Cr was 0.32 (IQR, 0.11–0.94) g/g (Table 1). Spearman correlations between baseline plasma and urine biomarker levels and characteristics of the patient population are presented in Supplemental Table 2. The median LVMI Z-score was 0.15 (IQR, −0.74 to 1.04), and the overall prevalence of LVH was 12% (*n*=59) (Figure 1).

**Table 1.** Baseline characteristics by type of CKD

Characteristic	Overall (*N*=504)	Nonglomerular (*n*=348)	Glomerular (*n*=156)
Age, yr	12 (8–15)	10 (7–14)	14 (11–16)
Male, % (*n*)	62 (311)	64 (223)	56 (88)
Black, % (*n*)	19 (94)	15 (51)	28 (43)
Hispanic, % (*n*)	13 (67)	13 (46)	14 (21)
CKD time, yr	8 (4–12)	9 (6–13)	3 (1–6)
Height Z-score	−0.5 (−1.3 to 0.3)	−0.6 (−1.4 to 0.1)	−0.1 (−1 to 0.7)
Weight Z-score	0.1 (−0.7 to 1.0)	−0.1 (−0.9 to 0.7)	0.7 (−0.2 to 1.6)
BMI Z-score	0.4 (−0.3 to 1.3)	0.2 (−0.4 to 1.1)	0.9 (0.2–1.6)
eGFR, ml/min/1.73 m^2^	54 (41–68)	51 (39–63)	63 (48–79)
**CKD stage, % (*n*)**			
1 (≥60 ml/min per 1.73 m^2^)	5 (24)	2 (7)	11 (17)
2 (≥60 ml/min per 1.73 m^2^)	33 (164)	27 (94)	45 (70)
3a (45–60 ml/min per 1.73 m^2^)	30 (153)	34 (117)	23 (36)
3b (30 to <45 ml/min per 1.73 m^2^)	25 (125)	29 (101)	15 (24)
4–5 (<30 ml/min per 1.73 m^2^)	8 (38)	8 (29)	6 (9)
Blood urea nitrogen, mg/dl	24 (17–31)	24 (17.5–32)	22 (16–30)
HTN, % (*n*)	17 (85)	19 (65)	13 (20)
Hemoglobin, g/dl	12.7 (11.7–13.7)	12.8 (11.8–13.7)	12.4 (11.6–13.6)
Urine protein-to-creatinine, g/g	0.32 (0.11–0.94)	0.24 (0.1–0.67)	0.62 (0.21–1.62)
RAAS inhibitor, % (*n*)	57 (285)	45 (155)	83 (130)
Anti-HTN drugs, % (*n*)	10 (52)	10 (36)	10 (16)
Premature birth (<36 wk), % (*n*)	11 (52)	11 (38)	9 (14)

BMI, body mass index; HTN, hypertension; RAAS, renin–angiotensin–aldosterone system.

### Plasma and Urine Biomarkers Associated with LVH Risk and LVMI Z-Score

We examined the associations of plasma and urine biomarker concentrations with prevalent LVH. In the unadjusted model, the following plasma and urine biomarker levels were associated with LVH: plasma KIM-1 (prevalence ratio [PR] per log_2_: 1.37, 95% confidence interval [CI], 1.17 to 1.60), plasma suPAR (PR: 1.61, 95% CI, 1.09 to 2.38), urine KIM-1 (PR: 1.28, 95% CI, 1.11 to 1.48), urine MCP-1 (PR: 1.29, 95% CI, 1.15 to 1.46), and urine EGF (PR: 0.78, 95% CI, 0.65 to 0.94). In the fully adjusted model accounting for demographics and all clinical characteristics, including eGFR and urine Pr/Cr, only two-fold higher levels of plasma KIM-1 (PR: 1.27, 95% CI, 1.02 to 1.58), urine KIM-1 (PR: 1.21, 95% CI, 1.02 to 1.44), and urine MCP-1 (PR: 1.18, 95% CI, 1.06 to 2.92) were positively associated with LVH prevalence. Two-fold higher levels of urine alpha-1m (PR: 0.90, 95% CI, 0.82 to 0.99) were associated with a lower prevalence of LVH (Table 2).

**Table 2.** Plasma and urine biomarker prevalence ratio from first visit for prevalence of left ventricular hypertrophy in CKD in children cohort

Biomarker, Per Doubling	Unadjusted	Adjusted Model (Plus Age, Gender, Race, BMI, HTN Status, GD)					Full Adjusted Model (Plus eGFR, Urine Pr/Cr)		
	PR	95% CI	*P* Value	PR	95% CI	*P* Value	PR	95% CI	*P* Value
**Plasma**									
KIM-1	1.37^a^	(1.17 to 1.60)^a^	<0.001^a^	1.39^a^	(1.17 to 1.65)^a^	<0.001^a^	1.27^a^	(1.02 to 1.58)^a^	0.031^a^
TNFR-1	1.24	(0.97 to 1.58)	0.085	1.36^a^	(1.09 to 1.71)^a^	0.007^a^	1.01	(0.66 to 1.54)	0.99
TNFR-2	1.43	(0.91 to 2.27)	0.123	1.53^a^	(1.01 to 2.30)^a^	0.045^a^	1.06	(0.62 to 1.82)	0.83
suPAR	1.61^a^	(1.09 to 2.38)^a^	0.016^a^	1.48^a^	(1.04 to 2.12)^a^	0.030^a^	1.05	(0.67 to 1.66)	0.83
**Urine**									
KIM-1	1.28^a^	(1.11 to 1.48)^a^	0.001^a^	1.29^a^	(1.10 to 1.50)^a^	0.002^a^	1.21^a^	(1.02 to 1.44)^a^	0.030^a^
MCP-1	1.29^a^	(1.15 to 1.46)^a^	<0.001^a^	1.25^a^	(1.10 to 1.41)^a^	<0.001^a^	1.18^a^	(1.04 to 1.34)^a^	0.012^a^
YKL-40	1.00	(0.97 to 1.04)	0.80	1.00	(0.97 to 1.04)	0.86	0.99	(0.96 to 1.02)	0.60
alpha-1m	1.01	(0.92 to 1.09)	0.91	1.03	(0.94 to 1.13)	0.55	0.90^a^	(0.82 to 0.99)^a^	0.026^a^
EGF	0.78^a^	(0.65 to 0.94)^a^	0.010^a^	0.71^a^	(0.57 to 0.88)^a^	0.002^a^	0.82	(0.59 to 1.15)	0.26

^a^Represents statistical significance.

Left ventricular hypertrophy is defined as left ventricular mass index ≥95th percentile for healthy children and adolescents. Left ventricular mass index is an index of left ventricular mass to height (mass [g]/height [m^2.7^]) to account for body size. Full model is adjusted for age, sex, race, glomerular diagnosis, body mass index, hypertension, urine protein-to-creatinine ratio, and baseline eGFR. Per doubling prevalence ratios are for a continuous log_2_ change in biomarker levels. BMI, body mass index; HTN, hypertension; GD, glomerular diagnosis; urine Pr/Cr, urine protein-to-creatinine ratio; PR, prevalence ratio; CI, confidence interval; KIM-1, kidney injury molecule-1; TNFR, tumor necrosis factor receptor; suPAR, soluble urokinase-type plasminogen activator receptor; MCP-1, monocyte chemoattractant protein-1; alpha-1m, alpha-1-microglobulin; EGF, epidermal growth factor.

Only urine YKL-40 demonstrated evidence of a significant difference in the effect estimates by glomerular disease etiology (Supplemental Table 3). Among those with nonglomerular disease, urine YKL-40 was associated with LVH in the unadjusted model (PR: 1.15, 95% CI, 1.03 to 1.28, interaction *P* value: 0.007), but not in those with glomerular disease (PR: 0.98, 95% CI, 0.95 to 1.01). However, after multivariable adjustment, effect estimates for urine YKL-40 in those with nonglomerular disease were attenuated and not significant (PR: 1.06, 95% CI, 0.98 to 1.13).

We also studied whether plasma and urine biomarkers were associated with LVMI Z-score. In the fully adjusted model, only plasma KIM-1 (*β*: 0.13, 95% CI, 0.03 to 0.22) was found to be significantly associated with LVMI Z-score (Table 3). There were no significant differences in the effect estimates for all the biomarkers and LVMI Z-score when stratified by glomerular versus nonglomerular etiology of CKD (interaction *P* value >0.05) (Supplemental Table 4).

**Table 3.** Plasma and urine biomarkers from first visit and left ventricular mass index Z-score from second visit

Biomarker, Per Doubling	Biomarker Alone			Adjusted Model (Plus Age, Gender, Race, BMI, HTN Status, GD)			Full Adjusted Model (Plus eGFR, Urine Pr/Cr)		
	*β*	95% CI	*P* Value	*β*	95% CI	*P* Value	β	95% CI	P Value
**Plasma**									
KIM-1	0.18^a^	(0.09 to 0.26)^a^	<0.001^a^	0.19^a^	(0.11 to 0.28)^a^	<0.001^a^	0.13^a^	(0.03 to 0.22)^a^	0.008^a^
TNFR-1	0.15^a^	(0.04 to 0.26)^a^	0.007^a^	0.21^a^	(0.09 to 0.32)^a^	<0.001^a^	−0.04	(−0.20 to 0.12)	0.64
TNFR-2	0.26^a^	(0.10 to 0.42)^a^	0.002^a^	0.29^a^	(0.13 to 0.45)^a^	<0.001^a^	0.02	(−0.18 to 0.23)	0.83
suPAR	0.30^a^	(0.13 to 0.47)^a^	0.001^a^	0.28^a^	(0.11 to 0.45)^a^	0.001^a^	0.01	(−0.19 to 0.22)	0.90
**Urine**									
KIM-1	0.09^a^	(0.02 to 0.17)^a^	0.016^a^	0.09^a^	(0.02 to 0.17)^a^	0.015^a^	0.07	(−0.02 to 0.15)	0.12
MCP-1	0.11^a^	(0.04 to 0.17)^a^	0.001^a^	0.09^a^	(0.02 to 0.15)^a^	0.010^a^	0.05	(−0.02 to 0.12)	0.14
YKL-40	−0.004	(−0.02 to 0.01)	0.57	−0.004	(−0.02 to 0.01)	0.52	−0.01	(−0.02 to 0.004)	0.15
alpha-1m	0.02	(−0.01 to 0.06)	0.23	0.04^a^	(0.001 to 0.07)^a^	0.047^a^	−0.03	(−0.08 to 0.02)	0.19
EGF	−0.15^a^	(−0.24 to −0.06)^a^	0.002^a^	−0.238^a^	(−0.34 to −0.14)^a^	<0.001^a^	−0.08	(−0.22 to 0.06)	0.27

^a^Represents statistical significance.

Left ventricular hypertrophy is defined as left ventricular mass index ≥95th percentile for healthy children and adolescents. Left ventricular mass index is an index of left ventricular mass to height (mass [g]/height [m^2.7^]) to account for body size. Full model is adjusted for age, sex, race, glomerular diagnosis, body mass index, hypertension, urine protein to creatinine ratio (Pr/Cr), and baseline eGFR. Per doubling prevalence ratios are for a continuous log_2_ change in biomarker levels. BMI, body mass index; HTN, hypertension; GD, glomerular diagnosis; urine Pr/Cr, urine protein-to-creatinine ratio; CI, confidence interval; KIM-1, kidney injury molecule-1; TNFR, tumor necrosis factor receptor; suPAR, soluble urokinase-type plasminogen activator receptor; MCP-1, monocyte chemoattractant protein-1; alpha-1m, alpha-1-microglobulin; EGF, epidermal growth factor.

## Discussion

In this study, we observed that higher concentrations of plasma and urine KIM-1, urine MCP-1, and lower concentrations of urine alpha-1m were independently associated with an increased prevalence of LVH, even after adjustment for eGFR and other known factors associated with LVH. Our cross-sectional analysis shows that biologically plausible biomarkers of kidney tubular injury, dysfunction, and inflammation may indicate the severity of kidney pathology and are associated with LVH in children with CKD.

An increase of tubular injury and inflammation in the kidney may affect the health of distant organs, such as the lungs and heart.^19^ Kidney tubular injury and inflammation can lead to activation of the renin angiotensin system, adrenergic system, and sympathetic nervous system and cause endothelial dysfunction, all of which may affect blood pressure regulation.^9^ As such, the tubular injury and inflammation may be contributing to the severity of kidney disease and HTN, both risk factors for LVH.

We found that higher plasma and urine KIM-1 levels were independently associated with higher LVH prevalence. KIM-1 is a membrane-bound protein which is expressed in the proximal tubule and plays a role in mediating phagocytosis for apoptotic and necrotic epithelial cells during kidney injury.^20^ Higher concentrations of urine KIM-1 have been associated with a decline of kidney function, progression of CKD, and progression of CVD.^21–23^ In a retrospective cohort study of older adults by Driver *et al.*, participants with the highest quartile for urine KIM-1 concentration were at higher risk for developing heart failure (Hazard Ratio, 1.32; 95% CI, 1.02 to 1.70), when compared with the lowest quartile.^24^ In another study by Ibrahim *et al.*, plasma KIM-1 concentrations were higher in those with coronary artery stenosis (0.04 ng/ml, IQR, 0.01–0.1) compared with those without (0.03 ng/ml, IQR, 0.01–0.05) (*P* < 0.001), and plasma KIM-1 levels also improved risk model discrimination for predicting coronary artery stenoses.^25^ Furthermore, in a case–control study, Kaddourah *et al.* observed that children with dilated cardiomyopathy had a higher concentration of urine KIM-1 (386 pg/mg, IQR, 248.7–596.6) as compared with age and sex-matched healthy controls (307.3 pg/mg, IQR, 182.8–432.5) (*P* = 0.02).^26^

We also observed that urine MCP-1 concentrations were associated with LVH prevalence. MCP-1 is a CC chemokine that is found in monocytes and tubular epithelial cells and plays a role in immunoregulation, inflammation, and fibrosis.^27,28^ During injury, MCP-1 enhances the inflammatory response by directing monocytes and macrophages to affected areas. In human and animal studies, urinary MCP-1 concentrations were found to be correlated with kidney damage.^29–32^ Elevated levels of MCP-1 have also been associated with CVD. In a study conducted by de Lemos *et al.* that included 2270 patients experiencing acute coronary syndromes, elevated levels of plasma MCP-1 were associated with an increased risk of myocardial infarction.^33^ In a study by Viedt *et al.*, it was demonstrated that MCP-1 promotes the proliferation of vascular smooth muscle cells, and this atherogenic process may be influential in CVD development.

In addition, we observed that higher urine alpha-1m is associated with lower LVH prevalence. Alpha-1m is a small protein which is primarily synthesized in the liver, filtered at the glomerulus, and is completely reabsorbed by the healthy proximal tubule. Urine alpha-1m is thus an indicator of tubular dysfunction, and increased levels of urine alpha-1m have been associated with incident CKD and progression of CKD.^34^ Urine alpha-1m has been examined in adult studies where higher levels have been shown to be associated with coronary artery disease, heart failure, and CVD incidence.^34–36^ In a retrospective observational study of adults with acute heart failure, Ishiwata *et al.* found that higher urine alpha-1m levels were associated with a higher left ventricular ejection fraction (*P* = 0.002).^36^ This previous research does not explain why higher alpha-1m concentrations were associated with a lower prevalence of LVH in our study. This was the opposite directionality observed with the other plasma and urine biomarkers and was not consistent with our original hypothesis since alpha-1m is a biomarker of proximal tubular dysfunction. The unexpected directionality suggests a mechanism not yet understood or could represent a chance finding. In addition, previous research of proximal tubular dysfunction and cardiac remodeling was completed mostly in adult populations, and age may modify this relationship. If confirmed, these findings would suggest that in a specific patient population and context, urine alpha-1m may identify a mechanistic pathway that could be targeted to limit LVH.

The strengths of our study include the use of validated biomarker assays in a centralized laboratory, standardized echocardiogram studies, and the number of participants in this pediatric CKD cohort. However, the results of our study should be interpreted in the context of several limitations. Owing to fewer echocardiograms being performed after the 1-year study visit, this research was limited to a cross-sectional analysis as opposed to the evaluation of longitudinal echocardiograms. In addition, our study does not measure biomarkers that may better capture the pathophysiology of myocardial injury, hypertrophy, and cardiac fibrosis. Finally, to develop more robust biomarkers of LVH with larger point estimates, proteins specific to cardiac pathophysiology will need to be evaluated.

Despite its limitations, our study is the largest prospective pediatric cohort study to assess specific biomarkers of tubular injury and inflammation and their relationship to LVH, and as such, we were able to evaluate LVH in a heterogenous pediatric cohort with multiple diagnoses. Previous studies have established associations between kidney biomarkers, such as serum creatinine and plasma FGF23, and LVH in children experiencing CKD. However, serum creatinine alone does not adequately estimate kidney function in children, and FGF23 is not an excellent predictor of LVH in children with advanced CKD.^10,18^ Given the frequency of LVH in the CKD population and the effect of LVH on cardiovascular health, it is in turn crucial to discover additional biomarkers of LVH so we can better inform our understanding of the relationship between CKD and LVH and the pathophysiology of LVH. Biomarkers may not only be used to routinely and noninvasively identify those children at highest risk of LVH, but they ultimately may serve as therapeutic targets as well.

In conclusion, we demonstrated that plasma KIM-1, urine KIM-1, MCP-1, and alpha-1m were independently associated with prevalent LVH in children with CKD. Notably, the strength of association between these biomarkers and LVH was preserved despite adjustment for eGFR and urine Pr/Cr. Because previous research has established the association between KIM-1, MCP-1, and alpha-1m with CKD progression, our results suggest the presence of shared mechanisms leading to the progression of CKD and the development of LVH. Future research is needed to better understand how tubular health, injury, dysfunction, and inflammation contribute to LVH and cardiovascular health.

## Supplementary Material

SUPPLEMENTARY MATERIAL

## Data Availability

Data in this manuscript were collected by the Chronic Kidney Disease in children prospective cohort study (CKiD) with clinical coordinating centers (Principal Investigators) at Children's Mercy Hospital and the University of Missouri–Kansas City (Bradley Warady, MD) and Children's Hospital of Philadelphia (Susan Furth, MD, PhD), Central Biochemistry Laboratory (George Schwartz, MD) at the University of Rochester Medical Center, and data coordinating center (Alvaro Muñoz, PhD) at the Johns Hopkins Bloomberg School of Public Health. The CKiD study is funded by the National Institute of Diabetes and Digestive and Kidney Diseases, with additional funding from the National Institute of Child Health and Human Development, and the National Heart, Lung, and Blood Institute (U01-DK-66143, U01-DK-66174, U01DK-082194, U01-DK-66116). The CKiD website is located at http://www.statepi.jhsph.edu/ckid. The biomarker analyses were performed in the CKD Biomarker consortium Central Laboratory (Joseph Bonventre, MD, PhD).

## Supplemental Material

Supplemental Table 1. Biomarker intra-assay and interassay coefficients of variation.

Supplemental Table 2. Spearman correlations of plasma and urine biomarkers and baseline characteristics.

Supplemental Table 3. Biomarker concentrations from first visit and prevalence of LVH by glomerular versus nonglomerular diagnosis.

Supplemental Table 4. Biomarkers and LVMI Z-score stratified by glomerular versus nonglomerular diagnosis.
