# Supplementary material for: Associations of Biomarkers of Kidney Tubule Health, Injury, and Inflammation with Left Ventricular Hypertrophy in Children with CKD
Source: Kidney360. 2023 Jun 12;4(8):1039–47. doi: 10.34067/KID.0000000000000183 (PMC10476681; doi:10.34067/KID.0000000000000183)
Supplement: SUPPLEMENTARY MATERIAL [file kidney360-4-1039-s001.pdf]

Supplemental Table 1. Biomarker intra- and inter-assay coefficients of variation

	Plasma KIM-1	Plasma MCP-1	Plasma TNFR-1	Plasma TNFR-2	Plasma suPAR	Plasma YKL-40	Urine EGF	Urine KIM-1	Urine MCP-1	Urine YKL-40
Intra-assay CV	5.6	4.0	7.2	5.4	8.3	3.3	5.1	4.5	3.2	4.7
Inter-assay CV	6.3	5.1	8.5	7.3	8.4	5.2	9.2	7.8	8.6	9.1

KIM-1, kidney injury molecule-1. TNFR, tumor necrosis factor receptor. suPAR, soluble urokinase-type plasminogen activator receptor. MCP-1, monocyte chemoattractant protein-1, EGF, epidermal growth factor.

Supplemental Table 2. Spearman Correlations of Plasma and Urine Biomarkers and Baseline Characteristics

	P KIM-1	P TNFR-1	P TNFR-2	P suPAR	U KIM-1	U MCP-1	U EGF	U YKL-40	U alpha-1m	eGFR	U Pr/Cr	Age at Entry
P KIM-1	1.0000											
P TNFR-1	0.3401	1.0000										
P TNFR-2	0.2978	0.8482	1.0000									
P suPAR	0.2809	0.6612	0.6186	1.0000								
U KIM-1	0.5902	0.2433	0.2625	0.2094	1.0000							
U MCP-1	0.4849	0.2022	0.2311	0.2372	0.6405	1.0000						
U EGF	-0.3656	-0.5741	-0.4581	-0.4077	-0.1538	-0.1377	1.0000					
U YKL-40	0.1197	0.3134	0.2861	0.3228	0.2404	0.3363	0.0040	1.0000				
U alpha-1m	0.1316	0.4473	0.4193	0.4391	0.1687	0.2513	-0.1436	0.6987	1.0000			
eGFR	-0.1020	-0.6694	-0.5565	-0.5350	-0.0057	-0.0270	0.5038	-0.4031	-0.5925	1.0000		
U Pr/Cr	0.4148	0.3094	0.3075	0.2599	0.4755	0.4639	-0.2418	0.3501	0.4223	-0.2280	1.0000	
Age at Entry	0.1676	0.0440	-0.0492	0.0020	0.1215	0.0709	-0.4252	-0.2657	-0.2203	0.0684	0.1793	1.0000

Plasma, P. Urine, U. Protein to creatinine ratio, Pr/CR. KIM-1, kidney injury molecule-1. TNFR, tumor necrosis factor receptor. suPAR, soluble urokinase-type plasminogen activator receptor. MCP-1, monocyte chemoattractant protein-1. Alpha-1m, alpha-1-microglobulin. EGF, epidermal growth factor.

Supplemental Table 3. Biomarker Concentrations from First Visit and Prevalence of LVH by Glomerular vs. Non-Glomerular Diagnosis

Biomarker, per doubling	Biomarker alone			Adjusted Model (plus age, gender, race, BMI, HTN status, GD)			Full Adjusted Model (plus eGFR, Urine Pr/Cr)		
	PR	95% CI	P-value	PR	95% CI	P-value	PR	95% CI	P-value
**Non-glomerular (n=348, 37 events)**									
Plasma									
KIM-1	**1.36**	**(1.16, 1.60)**	**0.000**	**1.63**	**(1.30, 2.06)**	**<.0001**	**1.48**	**(1.12, 1.97)**	**0.006**
TNFR-1	**1.28**	**(1.00, 1.63)**	**0.046**	**1.45**	**(1.04, 2.02)**	**0.030**	1.07	(0.65, 1.77)	0.784
TNFR-2	1.48	(0.94, 2.34)	0.094	**1.91**	**(1.10, 3.32)**	**0.022**	1.29	(0.69, 2.42)	0.417
suPAR	**1.65**	**(1.13, 2.39)**	**0.009**	1.47	(0.93, 2.33)	0.099	1.06	(0.62, 1.78)	0.841
Urine									
KIM-1	**1.29**	**(1.12, 1.48)**	**0.000**	**1.37**	**(1.07, 1.75)**	**0.013**	**1.27**	**(0.99, 1.62)**	**0.057**
MCP-1	**1.33**	**(1.15, 1.55)**	**0.000**	1.28	(0.99, 1.65)	0.059	1.14	(0.87, 1.49)	0.350
YKL-40	**1.15**	**(1.03, 1.28)**	**0.011**	1.10	(0.99, 1.21)	0.070	1.06	(0.98, 1.13)	0.128
alpha-1m	1.03	(0.93, 1.15)	0.590	1.16	(0.96, 1.39)	0.116	0.97	(0.81, 1.15)	0.722
EGF	**0.80**	**(0.66, 0.97)**	**0.023**	**0.68**	**(0.52, 0.88)**	**0.004**	0.80	(0.57, 1.13)	0.209
**Glomerular (n=156, 22 events)**									
Plasma									
KIM-1	**1.36**	**(1.14, 1.63)**	**0.001**	1.18	(0.91, 1.52)	0.204	1.10	(0.83, 1.45)	0.529
TNFR-1	1.24	(0.98, 1.57)	0.075	1.30	(0.96, 1.78)	0.094	0.96	(0.61, 1.53)	0.876
TNFR-2	1.45	(0.92, 2.27)	0.110	1.30	(0.73, 2.31)	0.380	0.94	(0.51, 1.74)	0.846
suPAR	**1.60**	**(1.11, 2.32)**	**0.012**	1.49	(0.87, 2.57)	0.148	1.05	(0.57, 1.94)	0.876
Urine									
KIM-1	**1.25**	**(1.06, 1.47)**	**0.009**	**1.22**	**(1.01, 1.48)**	**0.039**	1.17	(0.94, 1.45)	0.151
MCP-1	**1.26**	**(1.10, 1.45)**	**0.001**	**1.23**	**(1.08, 1.41)**	**0.003**	**1.20**	**(1.04, 1.37)**	**0.011**
YKL-40	0.98	(0.95, 1.01)	0.269	0.98	(0.95, 1.02)	0.326	0.98	(0.95, 1.01)	0.132
alpha-1m	0.99	(0.90, 1.09)	0.850	0.98	(0.88, 1.09)	0.706	0.88	(0.81, 0.97)	0.010
EGF	**0.78**	**(0.64, 0.94)**	**0.009**	0.74	(0.54, 1.01)	0.054	0.85	(0.55, 1.30)	0.450
**Interaction P-value**									
Plasma									
KIM-1			0.891			0.064			0.092
TNFR-1			0.163			0.657			0.655
TNFR-2			0.171			0.346			0.386
suPAR			0.178			0.968			0.990
Urine									
KIM-1			0.546			0.481			0.598
MCP-1			0.482			0.816			0.734
YKL-40			0.007			0.041			0.041
alpha-1m			0.519			0.129			0.326
EGF			0.523			0.672			0.782

Left ventricular hypertrophy (LVH) is defined as LVMI ≥95thpercentile for healthy children and adolescents. Left ventricular mass index (LVMI) is an index of left ventricular mass to height (mass [g]/height $[m]^{2.7}$) to account for body size. Full model is adjusted for age, gender, race, glomerular diagnosis (GD), body mass index, hypertension (HTN), urine protein to creatinine ratio (Pr/Cr), and baseline eGFR. KIM-1, kidney injury molecule-1. TNFR, tumor necrosis factor receptor. suPAR, soluble urokinase-type plasminogen activator receptor. MCP-1, monocyte chemoattractant protein-1. Alpha-1m, alpha-1-microglobulin. EGF, epidermal growth factor., PR, prevalence ratio. Per doubling PRs are for a continuous log_2 change in biomarker levels.

Supplemental Table 4. Biomarkers and LVMI Z-score stratified by Glomerular vs. Non-Glomerular diagnosis									
Biomarker, per doubling	Biomarker alone			Adjusted Model (plus age, gender, race, BMI, HTN status, GD)			Full Adjusted Model (plus eGFR, Pr/Cr)		
	β	95% CI	P-value	β	95% CI	P-value	β	95% CI	P-value
**Non-glomerular (n=348, 37 events)**									
Plasma									
KIM-1	0.17	(0.06, 0.29)	0.004	0.20	(0.09, 0.32)	0.001	0.14	(0.02, 0.26)	0.019
TNFR-1	0.18	(0.04, 0.33)	0.011	0.24	(0.10, 0.38)	0.001	0.00	(-0.17, 0.18)	0.976
TNFR-2	0.27	(0.06, 0.47)	0.010	0.30	(0.10, 0.50)	0.003	0.06	(-0.17, 0.28)	0.628
suPAR	0.32	(0.10, 0.55)	0.005	0.34	(0.12, 0.56)	0.003	0.08	(-0.16, 0.33)	0.493
Urine									
KIM-1	0.07	(-0.03, 0.17)	0.158	0.09	(-0.01, 0.19)	0.085	0.06	(-0.04, 0.16)	0.272
MCP-1	0.10	(0.01, 0.18)	0.021	0.08	(0.004, 0.16)	0.038	0.06	(-0.02, 0.14)	0.126
YKL-40	-0.01	(-0.02, 0.01)	0.276	-0.01	(-0.02, 0.01)	0.380	-0.01	(-0.03, 0.00)	0.124
alpha-1m	0.01	(-0.03, 0.06)	0.657	0.03	(-0.01, 0.07)	0.196	-0.03	(-0.08, 0.02)	0.209
EGF	-0.14	(-0.26, -0.02)	0.018	-0.26	(-0.39, -0.14)	0.000	-0.11	(-0.27, 0.04)	0.160
**Glomerular (n=156, 22 events)**									
Plasma									
KIM-1	0.19	(0.07, 0.32)	0.003	0.18	(0.06, 0.30)	0.005	0.11	(-0.02, 0.24)	0.108
TNFR-1	0.13	(-0.05, 0.31)	0.171	0.15	(-0.03, 0.33)	0.096	-0.11	(-0.32, 0.10)	0.301
TNFR-2	0.27	(-0.002, 0.55)	0.051	0.27	(0.001, 0.54)	0.049	-0.05	(-0.36, 0.25)	0.727
suPAR	0.06	(-0.01, 0.12)	0.077	0.05	(-0.01, 0.11)	0.109	-0.03	(-0.09, 0.04)	0.461
Urine									
KIM-1	0.30	(0.03, 0.56)	0.027	0.20	(-0.06, 0.46)	0.138	-0.09	(-0.38, 0.19)	0.522
MCP-1	0.13	(0.01, 0.26)	0.032	0.09	(-0.03, 0.22)	0.126	0.03	(-0.10, 0.15)	0.673
YKL-40	0.01	(-0.02, 0.04)	0.393	0.00	(-0.02, 0.03)	0.815	0.00	(-0.03, 0.02)	0.786
alpha-1m	-0.16	(-0.32, 0.00)	0.057	-0.20	(-0.36, -0.04)	0.015	-0.02	(-0.21, 0.18)	0.864
EGF	0.11	(-0.01, 0.23)	0.062	0.10	(-0.01, 0.21)	0.083	0.08	(-0.04, 0.20)	0.209
**Interaction P-value**									
Plasma									
KIM-1			0.837			0.784			0.682
TNFR-1			0.612			0.421			0.303
TNFR-2			0.967			0.848			0.508
suPAR			0.899			0.417			0.295
Urine									
KIM-1			0.629			0.857			0.792
MCP-1			0.611			0.891			0.622
YKL-40			0.200			0.525			0.586
alpha-1m			0.229			0.567			0.852
EGF			0.881			0.515			0.331

Left ventricular hypertrophy (LVH) is defined as LVMI ≥95thpercentile for healthy children and adolescents. Left ventricular mass index (LVMI) is an index of left ventricular mass to height (mass [g]/height [m]$^{2.7}$) to account for body size. Full model is adjusted for age, gender, race, glomerular diagnosis (GD), body mass index, hypertension (HTN), urine protein *to creatinine ratio (Pr/Cr), and baseline eGFR. KIM-1, kidney injury molecule-1. TNFR, tumor necrosis factor receptor. suPAR, soluble urokinase-type plasminogen activator receptor. MCP-1, monocyte chemoattractant protein-1. Alpha-1m, alpha-1-microglobulin. EGF, epidermal growth factor., PR, prevalence ratio. Per doubling PRs are for a continuous $\log_2$ change in biomarker levels.*
